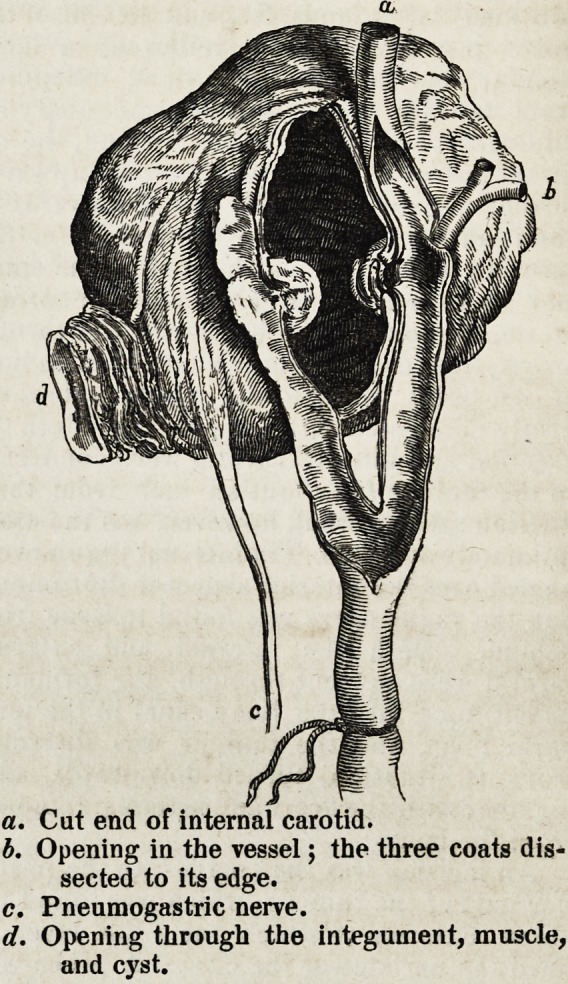# Medico-Chirurgical Transactions

**Published:** 1843-01

**Authors:** 


### Art. XIII.

*Medico-Chirurgical Transactions.* Vol. XXV.—*London*, 1842. 8vo, pp. 319. With 7 Plates.

The papers in this volume are for the most part short, and scarcely open to criticism; but as they offer many points of considerable interest, we shall, according to custom, present our readers with a brief analysis of them.

1. *A case of cyanosis, depending upon transposition of the aorta and pulmonary artery*, by Dr. Walshe. It is peculiarly interesting from the extreme rarity of the malformation, and is drawn up with a degree of precision and minuteness that is almost as seldom met with. The subject was a male infant, aged ten months. The leaden hue of the integuments was general, but the discoloration was particularly deep at the toes and extremities of the fingers, and also at the upper lip and inner canthi of the eyes. The child died in a paroxysm of dyspnœa. After death the blue discoloration greatly diminished, disappearing altogether from the lip and canthi. Upon examination the position of the heart in the chest was found natural, but the apex was slightly twisted to the left, and was formed by the extremity of the right ventricle. The right half of the organ lay anterior to the left. The aorta rose from the right, and the pulmonary artery from the left ventricle. There was no communication between these two vessels, saving by the ductus arteriosus. The aorta gave off two subclavian and two carotid arteries from the upper border of the arch, and the two coronary arteries arose in the usual way. The ductus arteriosus opened into the aorta exactly opposite the origin of the left subclavian. The venæ cavæ entered the right auricle in the ordinary manner. The right auriculo-ventricular orifice had a mitral valve, and the left a tricuspid. The foramen ovale was perfectly open, and the right side of the heart had walls of a much greater thickness than the left. The whole organ was hypertrophous. The viscera generally, and the liver in particular, were larger than natural. The muscles and adipose system were defectively nourished, but not in an extraordinary degree. It is, therefore, quite evident that the nutrition of these parts may be carried on by almost entirely un-oxygenized blood, (for the quantity of red blood circulating in the arterial system must have been extremely small,) without any important deviation from the normal state, *when such a condition has existed from birth.* We strongly recommend a perusal of the paper.

II. *Case of aneurism of the ascending aorta, bursting into the right ventricle,* by Mr. Beck. Upon examination after death, (which was *not* sudden,) the heart was found hypertrophous. The right sinus of Valsalva was enlarged, and presented a round open communication between the aorta and the right ventricle, sufficiently large to admit the end of the little finger. The lining membrane of the right ventricle was of a white colour, and somewhat thickened; the valves were little changed from their natural state, but immediately beneath them lay the collapsed sac of an aneurism, resembling the end of a finger of a glove, about three-quarters of an inch in length, and having at its extremity a large ragged opening, with two small orifices at the side, the edges of all being worn and rounded, as if the blood had passed through them for some time. No coagulum was found in the sac. Immediately at the base of the sac there was a communication, the size of a goosequill, between the two ventricles.

The patient had been unable at any time to run or walk quickly any distance, without suffering from violent palpitation; but the severe symptoms, which terminated in death, did not commence until about five years before his decease. During life a continued and very superficial sawing sound, with tremor, was heard; it was loudest after the second sound, and was most distinct at the base of the heart near the sternum.

III. *On the structure and functions of the human placenta,* with a plate, by J. Dalrymple, Esq. The observations of this gentleman are chiefly corroborative of the description given by Weber. According to his views, the whole mass of the placenta is made up of innumerable ramifications of the umbilical arteries, terminating in beautifully coiled and convoluted capillaries, which form tufts or bouquets at various intervals, and finally become continuous with the umbilical vein. The vessels, and the tufts formed by the capillaries, are inclosed in prolongations of the chorion. The tufts are made up of villi, and each villus contains one tortuous capillary. The interstices between the vascular divisions and subdivisions are everywhere free, and communicate with each other. The supposed maternal cells of the placenta do not exist. Mr. Dalrymple differs from Dr. Reid in some respects. He nowhere observed the arteries and veins so closely bound together as to constitute one undivided though really double vessel, and he believes the "blunt extremities" adverted to by that gentleman to be what he has called "villi." He does not appear to have noticed the reflexion of the inner membrane of the maternal venous system upon the vessels of the embryo, which constitutes so interesting a portion of Dr. Reid's discovery. (See Br. and For. Med. Rev. vol. XI. p. 540.)

IV. *On the relation between the symmetry and the diseases of the body*, by JAMES PAGET.

X. *On diseases which affect corresponding parts of the body in a symmetrical manner*, by W. BUDD, M.D.

We shall throw together our notices of these two papers, because the objects at which the writers of both aim are identical, viz. the proof "that it is a law of the animal economy, that, when uninfluenced by disturbing causes, all general or constitutional diseases affect equally and similarly the corresponding parts of the two sides of the body." And in attempting to make our readers acquainted with what is yet known respecting this very curious subject, we shall first lay before them a few specimens of the kind of facts which serve as the basis of this opinion, and then expose to view the respective theories which our authors have formed to explain the occurrence of such phenomena.

It has been frequently observed, that, when a joint has been found diseased in one limb, the corresponding joint in the other limb has presented a similar affection. Of this Mr. Paget narrates some remarkable instances. In the body of a woman fifty-one years old, in both elbow-joints an irregularly triangular portion of cartilage had been removed from the middle of the great sigmoid cavity of the ulna, and into each of these spaces there had grown a process of synovial membrane and fat, which accurately fitted into it. Above each of these larger ulcerations there was a smaller one. In the two knee-joints of a woman of seventy the cartilages of the patella, femur, and head of tibia, were affected with the fibrous degeneration in precisely the same extent and degree, and in each the edges of the semilunar cartilages were similarly and equally affected by the same disease. Moreover, on each outer condyle there was a spot of exactly the same form and size, from which the cartilage was completely removed, and where the exposed and hardened bone formed a shallow depression, into which a corresponding elevation on the top of each tibia accurately fitted; and, in a still more striking instance, both hip-joints of a woman aged sixty-eight, who died of general dropsy, presented the following appearances: To the head of each femur was attached a similar very slender shred of fibrous tissue, the remnant of the ligamentum teres; on each there were similar small spots, from which the cartilage had been removed; and, still more, on the corresponding part of each neck of the femur, there was a spot from which the investing fibrous tissue had been absorbed by ulceration, leaving an aperture into which an irregular elevation of bone had grown, the resemblance being so close that the naked eye could scarcely detect any difference.

We have a very analogous class of facts in the distortions produced by gout and rheumatism, which are often remarkably symmetrical, and of which a particularly distinct example is figured by Dr. Budd ; in the appearances of divers congenital malformations ; and in the cartilaginous growths to which the bones of the hands and feet are liable. Ulcerations of the cornea from defective nutrition, syphilitic diseases of the eye, the phenomena of metastasis, the rapid passage of inflammation of the tonsils and Schneiderian membrane from one side to the other, and the distribution of atheromatous patches in corresponding parts of the arterial system, as shown by Bizot, are further examples of the same symmetrical tendency. But by far the most numerous instances are to be drawn from the extensive catalogue of skin diseases, in many of which the eruption, on corresponding parts of the opposite limbs, presents an almost perfect similarity both in pattern and the number of spots, (vide pl. III).

Such being the nature of the evidence for the existence of this law, let us now direct our attention to the explanations of the phenomena adopted by our authors, and here we shall observe a close degree of resemblance.

Mr. Paget believes that there are at least three different conditions in which diseased changes are symmetrical, viz. 1. When these changes are the result of the gradual degeneration of the tissues in the course of time, or after their functions have ceased, or when, through some general disorder in the economy, the whole body fails of being duly nourished. Such are emaciation, the changes of old age, &c. 2. They are the result of a morbid condition of the blood, in which some new material bears a peculiar chemical or organic relation to the whole or a part of some symmetrically-arranged tissue or organ, so that when they come in contact, the mode of nutrition in the tissue is altered, or the new material is deposited in it. And the changes are general or local, according as the similarity of the parts is perfect or partial. To this class belong, rheumatism, gout, scrofula, cancer, &c. 3. They result from metastasis, either with or without a morbid condition of the blood. (p. 40).

Dr. Budd divides the whole into two principal groups, viz. 1, Deviations arising from original fault in the solids, as symmetrical monstrosities ; and 2, Those which originate in morbid states of the blood, which comprise by far the greater number, and may be subdivided into those which arise from morbid matters of special kind in that fluid, and those which depend on deficiency of its natural ingredients. (p. 161.) When morbid matter exists, he believes it is detained in the seat of each individual lesion, and is there held in affinity with the part affected, this affinity being so elective, that the symmetrical or analogous parts of opposite regions of the frame are singled out by it, to the exclusion of all others, however like to these in outward appearance.

The disturbing influences which so often prevent their peculiar effects are, according to Dr. Budd, febrile movement, lesion, or other material cause of organic change, and variations in the amount of the morbid matter itself.

It will be seen at once that there is little real difference here, and that both have to a certain extent adopted a modified humoral pathology, as essential for the elucidation of otherwise inexplicable phenomena, in which we believe they have adopted the most soundly philosophical views. We cannot forbear quoting one example brought forward by Dr. Budd, because it satisfactorily illustrates the production of symmetrical disease by the agency of a substance introduced from without. A case occurred in King's College Hospital, in which the free administration of iodide of potassium was followed on the fourth day by an extensive erythematous eruption, the patches being distributed on the limbs and trunk in a perfectly symmetrical manner. (p. 112.)

It would be premature to pronounce decisively upon a subject which is only just beginning to be opened up, but we have no hesitation in recording the favorable impression which the perusal of these two very interesting papers has produced upon our minds; and we particularly recommend all who may be anxious to enter upon a most inviting field of investigation, to study with care the very elaborate production of Dr. Budd, which will serve them as an admirable guide.

v. *Case of extensive disease of the pancreas*, by J. A. Wilson, m.d. The subject of this case was a male, aged forty-one, who had been a free liver and had drunk much. His symptoms were, constant pain in the epigastrium of long continuance, and occasional paroxysms of severe agony. The suffering was greatest after taking food, and when he was in the recumbent posture. The paroxysms were accompanied by headach, giddiness, and sickness, and he frequently vomited blood. The fatal event was ushered in by a peculiarly severe attack of the epigastric pain, with shivering, intense headach and sickness. Maniacal delirium supervened, and was succeeded by complete coma. No organic disease could be detected during life, but upon examination after death the pancreas was found of smaller size than natural, and unusually hard in its texture, and its ducts were universally filled with a compact, white, earthy deposit, which on analysis was found to consist of nearly pure carbonate of lime, with a fibrinous nucleus of animal matter. The spleen fell into a grumous pulp under slight pressure; the liver was pale, soft, and friable, and the stomach and kidneys were healthy. This is an extreme case of a very rare form of disease, and is well deserving of notice. Our present knowledge of the functions of the pancreas is anything but satisfactory.

vi. *Remarks on typhus fever*, by J. Bostock, m.d. This short, but well-written paper, is the record of the views which Dr. Bostock has been led to adopt respecting the different forms of typhus, or perhaps more correctly, of continued fever, their mode of propagation and their treatment. It is the result of a long and extensive experience, and as such merits attention; but we do not find anything sufficiently novel to demand a place in these passing remarks.

vii. *Cases of laryngitis relieved by operation*, by John Wilson, m.d. Four cases are related; in two the operation was successful, in the others it failed to effect a cure. Their chief value consists in showing, that if the operation be attempted at all, it should be done at an early period, before the lungs are irremediably disorganized. The last case reads us another lesson upon the futility of surgical interference in croup.

viii. This is by the same author, and contains an account of the illness and death of an entire family, consisting of six persons, under very peculiar circumstances. The most prominent features in the sufferings of all were, general soreness of the fleshy parts, and also of the joints, exquisite sensibility of the skin, œdema, especially of the lower extremities, diarrhœa, the secretions being very much disordered, alkaline urine, great emaciation, (accompanied in some with a ravenous appetite,) and a teasing, dry cough, which was particularly severe in the children before the fatal event took place. The mother complained of a cankery, and one of the children of a metallic taste in the mouth, from which a watery fluid was discharged. The intellectual faculties were unimpaired. They appeared insensible to the influence of cold, for during severe nights of winter they would scarcely endure the covering of a single sheet.

On examination after death, the appearances of disease in the alimentary canal were altogether trifling; but in all, (excepting the mother, whose death was hastened by puerperal fever,) the lungs were extensively affected, their tissue being infiltrated with black blood. The parts thus disorganized presented the appearance of pulmonary apoplexy, with this exception, that they were not circumscribed by healthy lung, the transition from the condensed to the permeable portions being gradual. No poison could be detected by chemical analysis, and the cause of death was utterly unknown. The father was an Italian, a manufacturer of ultramarine. He did not live happily with his wife.

ix. A very interesting *case of congenital cataract*, which had existed for twenty-three years, and in which the patient acquired perfect vision after operation. It is related by the operator, Mr. Stafford. This is the longest period on record after which a cure was effected. The patient in Cheselden's case was thirteen years old, and in Mr. Ware's seven.

xi. *On cases of plague*, by M. Pezzoni, in a letter to Dr. Davy. The object of this paper is to prove that the plague is really contagious; and as the facts narrated appear to be authenticated in a satisfactory manner, it deserves a careful perusal.

xii. *Observations on tubercle of the brain in children*, by P. Hennis Green, m.b. The data, upon which these observations are founded, are drawn from the results of thirty cases, in which the symptoms were carefully observed during life, and the diseased appearances accurately noted after death. They are presented in a tabular form. Of the whole number, fourteen were boys, and sixteen girls. The ages varied from nineteen months to twelve years, the greatest number occurring between one and four years. In five cases there were no symptoms whatever of cerebral disorder; in three, headach was the only symptom during the chronic stage; in one deafness, and in one purulent discharge from the ear. In the remaining twenty cases the symptoms were more complicated.

In the course of the disease two stages may generally be recognized, a *chronic* and an *acute*. Dr. Green considers that there are three classes of symptoms presented during the chronic stage. In the *first*, the disease commences with headach, which, indeed, is the most common and characteristic of all the symptoms; it formed a prominent feature in seventeen of the twenty cases. It is most usually frontal, is occasionally associated with vomiting, and is followed by various lesions of sensibility or of muscular power. In the *second* class, the disease commences with convulsions or epilepsy, which gradually terminate in paralysis. (A very interesting case of this kind forms the subject of the next paper, No. xiii, narrated by Mr. Dunn.) In the *third* class, paralysis of one or more muscles, or organs of sense, is the first symptom observed.

The symptoms of the *acute* stage are varied, but are generally more or less allied to those of hydrocephalus or ramollisement.

In eleven of the whole number of cases the tubercles were seated in the cerebral hemispheres, in nine they existed in the cerebellum, in seven they were found in both the cerebrum and cerebellum, and in two in the cerebellum and pons varolii. In Mr. Dunn's case they occupied the *surface* of the right cerebral hemisphere.

XIV. *Case of stricture of the trachea*, by Mr. W. C. WORTHINGTON. The disease was of a very chronic character, and appeared to have a syphilitic origin. The most prominent symptoms during life were, the peculiar noise attendant upon inspiration, and the painful effort required for its accomplishment. The noise precisely resembled that which is made by an unsound horse, called a roarer. Each inspiration occupied ten seconds, and was accompanied by violent action of the muscles attached to the larynx. Vocalization was very imperfect, the sound of utterance being rough and hoarse. Upon inspection after death, (which was caused by suffocation from some particles of food,) a well-defined constriction, forming a complete stricture, was discovered just below the cricoid cartilage, the caliber of this portion not exceeding that of a crow-quill. When opened from behind, the trachea presented the following appearances: superficial cicatrices of a smooth and polished appearance, extending both below and above the stricture, entire absorption of the cartilaginous rings of the trachea, from about half an inch below the thyroid cartilage downwards to the extent of about three inches; constriction of this portion, the inner surface being quite smooth; dilatation of the trachea below, larynx sound.

XV. *Case of tumours in the head and face*, by Mr. ANCELL. The subject of this case was an unmarried female, aged fifty-two. The disease first appeared when she was about fourteen or fifteen years old. The greater part of the scalp and face was loaded with solid tumours, of different sizes. Those on the scalp were externally of a very florid colour, smooth, glassy, and denuded of hair; in shape they varied from a nearly globular to an irregular, flattened spheroidal form, with a tendency to assume a mamillated outline. Interspersed among them were a few perfectly round, and of a violet hue. Some of the tumours were sessile on broad bases; others were suspended by short, thick peduncles. One of these latter was removed, and when divided showed a smooth, shining, semi-transparent texture, of a very pale pinkish hue, and a nearly cartilaginous consistence; it appeared to be homogeneous, excepting that a few vessels ramified through it: the investing skin was much more vascular. Similar tumours were scattered over the face, but they were mixed with tubercles of a different nature. The layer of these had all the characters of lenticular tubercles, depending upon hypertrophy of the dermis, while most of the smaller ones were follicular elevations, such as accompany other cutaneous diseases. The tumours sometimes itched; they were painful when pinched, but were generally free from uneasiness. At one time a few were extirpated, and subsequently Mr. Bryant removed sixty at one sitting. They had then a different appearance; they did not approach a cartilaginous consistence, and on making a longitudinal incision, the contents were easily turned out. Within twelve months of the operation they were all reproduced.

About five months before she came under Mr. Ancell's care, the patient, who had enjoyed uninterrupted good health for a series of years, discovered something hard in the abdomen. The tumour was uneven in surface, and was situated in the right hypochondrium. Ascites supervened after a short period, and was followed by anasarca of the lower extremities, and she gradually sank from exhaustion.

After death the following appearances were found in the abdomen. The peritoneum was generally opaque, but with a shining surface. The parietal portion, and the lining of the diaphragm were studded with myriads of tumours, of various sizes. The fat of the great omentum was almost entirely absorbed, and its tissue was sprinkled over with numberless granules: some larger masses being interspersed, many of which were merely suspended by afferent and efferent blood-vessels, with a few shreds of cellular tissue. A very large mass, weighing about two pounds, was suspended from the anterior edge of the liver; it extended beneath the right lobe, displacing and pressing the gall-bladder downwards into Glisson's capsule. It was of an irregular ovoid form, with a nodulated surface, and a very firm texture. When divided, the tints of the cut surfaces were extremely varied, green and greenish-yellow predominating. In the centre it was nearly white, and almost cartilaginous; and there were distinct fibrous radii of irregular dimensions, proceeding from the centre towards the circumference. The remainder of its substance was made up of lobules, with an indistinctly cystiform aspect. Blood oozed on pressure from a good many red points, but the tumour could not be called highly vascular.

The disease appears to have been hereditary, but was confined to the females of the family, who where also remarkably prolific.

XVI. *Case of malformation of the heart*, by Theophilus Thompson, M.D. The right ventricle was divided into two cavities by an imperfect septum, composed of decussating and hypertrophied columnæ carneæ; some of which, separating from each other near the base of the ventricle, left an aperture of communication about one inch long, and half an inch broad. The circumference of the pulmonary artery exceeded that of the aorta by nearly an inch, and it had four perfect valves of equal size, a very rare, if not unique deviation from the natural structure.

XVII. *Case of petechial cowpox*, by Dr. Gregory. An apparently healthy female child was vaccinated from unexceptionable lymph, in five places on the left arm. On the 5th day, the mother observed that the arm was more inflamed in this child than in two others of her children who had been vaccinated at the same time, and from the same matter, and she also noticed some spots on the face. On the ninth day, when Dr. Gregory first saw the child, the outer portions of a large areolous circle had assumed a yellowish tint, while the inner portions were still of a dark mahogany colour. The vesicles themselves were jet black. There were numerous petechiæ over the body. On the left temple there was a very large extravasation of blood, owing to a slight bruise. There had been some bleeding from the left ear, and a few drops of blood had escaped from the nostril, but none was discharged by stool. The ecchymosed state of the arm and the petechiæ declined with the cowpox, and on the sixteenth day all hemorrhagic appearances had ceased. During the whole time the child was in perfect health.

XVIII. *On the ulceration of the duodenum after burns*, by Mr. CURLING. This is a very interesting paper, showing that in cases of severe burns, which are apparently advancing favorably towards recovery, a fatal result is often rapidly induced by acute ulceration of the duodenum, especially of that portion which passes round the head of the pancreas. Mr. Curling believes the glands of Brunner to be the seat of disease, the obstructed functions of the skin exciting them to undue action. Should such a state of things be suspected during life, he recommends the application of leeches to the corresponding part of the abdomen, the exhibition of hydrarg. c. creta with opium, and a very mild fluid diet.

XIX. *Cases of malformation of the heart*, by Dr. T. B. E. FLETCHER. These cases are interesting but to the morbid anatomist. Our limited space prevents our noticing them at large.

XX. *On tumours in the neck*, by B. PHILLIPS. The design of this communication is to show that there is a class of uni-or multi-locular encysted tumours in the neck, which contain a serous fluid, varying from a light yellow to a deep-coffee colour, and which are generally developed quite independently of the thyroid body, though in their course they may become intimately connected with it. They are generally developed at or after the middle period of life, are almost always of slow growth, and often attain a large size. Puncture and seton appear the best method of treatment. The cases are instructive and deserve attention.

---

*On a variety of False Aneurism.* By ROBERT LISTON, F.R.S., Surgeon to University College Hospital, Professor of Clinical Surgery in University College, &c. &c. (Read to the Royal Medical and Chirurgical Society, March 8th, 1842. Printed at the author's expense for distribution among the members.) 8vo, pp. 39.

Why this paper has been "printed at the author's expense, for distribution among the members" of the Royal Medical and Chirurgical Society, and not published in the Transactions of that body, we are at a loss to discover, and probably the Controlling Council might feel some little difficulty in returning a plain answer to the question. To our thinking, no narration more instructive to the practical surgeon, or interesting to the pathologist, has graced their pages for some years past; and so far as our readers are concerned, we take leave to remedy the omission in the aforesaid Transactions, by laying before them the leading points of the unjustly-slighted memoir.

In a puny boy aged nine,* a small swelling began to form immediately below the right ear, about two months before his admission into the North

* The age has been since stated to be 12.

London Hospital. It was fomented and poulticed, and increased gradually. Within three or four days of his admission its progress became more rapid and its shape irregular. He presented himself October 20th, —"Having a tumour at the angle of the jaw on the right side, extending backwards as far as the posterior border of the sterno-mastoid muscle, (the upper part of which was pushed forwards,) downwards to within an inch of the clavicle, and forwards to about half the length of the horizontal ramus of the lower jaw. It projected into the mouth between the arches of the palate, impeding in a great degree both respiration and deglutition. Its most prominent point was posteriorly and superiorly at the outer border of the sterno-mastoid. Indistinct fluctuation could be felt, and there was slight pulsation in it immediately over the carotid artery; but on grasping the sides of the tumour no pulsation could be discovered, nor could any be felt inside the mouth. Mr. Liston made a small puncture into the tumour under the impression that it contained matter; a gush of arterial blood followed the operation, and about four ounces were lost in a few seconds; the wound was closed by hare-lip pins and the twisted suture, and the bleeding thus checked. Mr. Liston determined to tie the carotid on the following day." (p. 5.)

Next day the carotid was tied, after a deep and difficult dissection, immediately above its origin from the innominata. The swelling decreased, and the case seemed to be advancing favorably. On the thirteenth day, however, bleeding occurred from the point of arterial deligation; and, by repetition, carried off the patient on the evening of the fifteenth day after the operation.

The account of the inspection after death we give almost entire:

"A small quantity of blood was found to be extravasated in the superficial cellular tissue around the puncture. The superficial fascia was now raised, and the sterno-mastoid, together with the other muscles of the neck, were dissected down. The incision which was made in placing the ligature on the artery was found to have divided the sterno-hyoid and thyroid muscles, as well as part of the sterno-mastoid. The tissues around it were consolidated by effused lymph. The parotid gland appeared to extend lower down into the neck than usual, and along the anterior edge of the sterno-mastoid muscle were situated three lymphatic glands, each enlarged to about the size of a small walnut; the lower one extended to within a quarter of an inch of the superior extremity of the incision. These, with several smaller glands, almost entirely filled up the superior part of the triangle, the trachea being somewhat to the left side, and the sterno-mastoid muscle to the right; the carotid artery in this situation was seen uncovered by the glands. The external jugular vein was found to cross the mastoid muscle in the ordinary situation, being about half an inch internal to the puncture, which was through the posterior fibres of the muscle. Just opposite to the angle of the jaw lay another enlarged lymphatic gland, somewhat larger than any that have been mentioned, and extending partly under the sterno-mastoid muscle. The whole of these parts were firmly matted together by a dense deposit of lymph. The clavicle was now cut through at about its external third, and the upper portion of the sternum was removed. The sterno-mastoid was then dissected upwards, but was found to be firmly adherent, at the point of puncture, to the tissue beneath. A portion was therefore left at this part, and the remainder of the muscle, into the substance of which a considerable quantity of blood had been effused, was removed. The enlarged glands, the sterno-hyoid and thyroid muscles, together with the deep fascia, were then removed, and the innominata, carotid, and sub-clavian arteries exposed. The ligature was found to have been placed close to the origin of the carotid from the innominata; it was not completely separated, a small portion of the external side of the artery still remaining entire. The proximal end of the vessel was quite open, and admitted a large-sized probe; there had been no attempt at the formation of a clot, or, if any had been formed, it must have been expelled with the blood. The distal end of the vessel was sealed by a firm coagulum, and around the situation of the ligature was a considerable deposit of firm lymph. The arteries arose from the arch in the usual manner. The dissection in the superior part of the neck was now proceeded with. The parotid and sub-maxillary glands, &c., and the side of the lower jaw having been removed, a large tumour was brought into view, extending from the side of the trachea and pharynx, outwards as far as, or a little beyond, the external border of the sterno-mastoid; upwards to the base of the skull, and downwards to about an inch below the bifurcation of the carotid; behind, it was limited by the spine and its muscles. Over the anterior surface of the tumour could be traced the carotid artery, free to within three quarters of an inch of its point of division, where it became firmly connected with the swelling. Both the external and internal carotid were connected to the tumour for about an inch from their division: the internal, however, was the more intimately attached. The internal jugular vein passed over the anterior aspect of the tumour, but the vagus nerve was found to issue from behind. Both the internal and external carotid were now cut through, the former at its entrance into the bony canal in the temporal bone, and the tumour was dissected from its situation, turned downwards, and, together with the heart and arteries, removed from the body.

An incision was then made into the posterior part of the tumour, which was found to contain a quantity of dark grumous blood, external to which was a thin layer of organized lymph which entirely lined the parietes of the cyst. A probe was passed down the internal carotid artery, and found to enter the cyst just opposite the division of the common carotid; this point was, however, obstructed, a mass of coagulable lymph almost entirely blocking up the entrance. The parietes of the tumour behind, were about a line or more in thickness, but on the outer side, on the aspect adjacent to the puncture, they were much thinner. They appeared to be composed of three layers differing in character, but organically connected together. The outermost layer consisted of condensed cellular tissue having portions of the surrounding structures attached to or imbedded in it. The middle layer, which was not so evident on the outer side of the tumour, was very dense and opaque, so that it appeared like a distinct white line on a section of the parietes of the tumour. The innermost layer was soft and pulpy, semi-transparent, and of a pale dirty red colour; its inner aspect, forming the inner surface of the cyst, was smooth, and for the most part even; but in some points, and especially at the posterior part of the cyst, it presented a fasciculated appearance like the interior of the auricles of the heart, or of a fasciculated bladder, only not so well marked; it consisted, as I have said, of a flaky lymph-like substance throughout. Opposite the outer part of the tumour (where its parietes were thinnest) this substance was flocculent and broken, and contained patches of a bright yellow colour; here it was not distinctly laminated, and it adhered rather firmly to the middle layer of the cyst, but in other situations it was easily separable into laminæ, and was not so firmly adherent. The cavity of the cyst contained a quantity of grumous blood. The common carotid and the internal and external carotid were firmly attached to the front of the tumour. The opening by which the cyst had communicated with the artery had been about three lines wide and two and a half lines long, and was situated at the bifurcation of the common carotid. It was now completely closed by a firm clot, in which no perforation was visible, so that the probe which had been passed down the internal carotid into the cavity, must have been forced either on one side of this clot or through it. On the side next the vessel, the surface of the clot was concave, broken in the centre, but smooth towards the circumference, where it was closely adherent to the margin of the opening in the carotid artery. The opposite side of the clot was convex, and projected into the cavity of the cyst. On making a vertical section through the artery, the walls of the tumour, and the clot, the latter was seen to be composed of fibrinous laminæ. The edges of the opening in the vessel were found to be well defined and slightly everted; the external coat of the artery was distinctly traced, and afterwards dissected from the middle coat quite up to the margin of the opening, where it terminated abruptly, not being reflected on to the outer surface of the tumour. The coats of the vessel showed not the slightest dilatation at the part where it was connected with the tumour." (pp. 8-14.)

*a.* Cut end of internal carotid.
*b.* Opening in the vessel; the three coats dissected to its edge.
*c.* Pneumogastric nerve.
*d.* Opening through the integument, muscle, and cyst.

It seems to us very plain that the foregoing case was of the exact nature which has been assigned to it by Mr. Liston. A chronic abscess gradually formed among the lymphatic glands in the upper angle of the neck of a boy, probably of a scrofulous habit original or acquired. That abscess had its original site in the immediate vicinity of the large vessels. As it slowly advanced they were displaced and compressed. For about two months the arterial coats, where most pressed upon, resisted the incitement to ulceration, by virtue of the inherent vital power with which they are so wisely endowed to that effect. But at last they gave way. The cavity of the abscess and the canal of the ulcerated artery now became continuous; and what had been, up to the period of arterial ulceration, the cyst of a chronic abscess, now became the sac of a false aneurism. This change took place three or four days previously to the boy's admission into the hospital; and was followed, as can readily be understood, by marked and sudden increase of the tumour. The true nature of the case, however, was not suspected. On examination all the characters of chronic abscess, latterly supposed to have sustained the supervention of an acute action, were apparent; those of an aneurismal complexion seemed but an ordinary simulation of that more grave disorder. An exploratory puncture disclosed the error of diagnosis. The disaster was instantly met, however, and that suitably. The only available treatment was put in force, and who dare honestly lay the blame of its unsuccessful issue on the surgeon?

That such was the actual train of events, a careful and impartial consideration of the case has fully convinced us. But we know that others think differently. And in the class of these "others," it is probable that a majority of the council of the Royal Medical and Chirurgical Society are to be found.

"It was ordinary aneurism," say some. We dissent, for the following reasons:

1. The patient's age is one at which spontaneous aneurism is very unlikely to occur. Take the latest authority on this point. "Seeing then that aneurism is at least negatively subject to certain laws, that it is not met with in animals, *that it is not met with in the human subject before the age of puberty*," &c. (Porter on Aneurism, p. 37.)

2. The opening in the vessel is small, and has all the appearance of recent formation by ulceration; its edges are ragged, abrupt, and well-defined; all the arterial coats stop at this aperture, and the cellular is not found spreading over the cyst; in other respects, likewise, the parietes of the cavity present the usual characters of the cyst of a chronic abscess in such a situation, thinning as usual towards the surface, and not those of an original aneurism, whether false or true; the inner aspect, "consisting of a flakey lymph-like substance throughout," looks like a pyogenic membrane, and does not at all resemble densely compacted fibrin of a laminated arrangement; there is no steatomatous or other degeneration of the arterial coats at the ulcerated point; no dilatation there. In brief, we find not one sign of spontaneous aneurism, in any of its known forms.

3. Had the tumour been originally an aneurism, the sudden accession to its growth, when it became rapidly enlarged, and of an irregular form, a few days before admission, could have proceeded but from one cause, viz., the diffuse form having supervened on the circumscribed. But that no such occurrence could have taken place is proved by the cyst being found continuous in its walls, which had at no point given way, but were merely thinning outwardly, as all cysts of abscesses are in the habit of doing, and by the absence of sanguineous infiltration in the surrounding tissue. "A small quantity of blood was found to be extravasated in the *superficial* cellular tissue, *around the puncture*," obviously caused by that puncture, and that was all.

But, say the upholders of aneurism, when driven from their pathological ground, "How do you reconcile an early *symptom*, which we have been assured existed in this case, with your ideas of its nature? It was at first a small tumour, compressible and capable of being made to disappear entirely by pressure." An abscess may be so, as well as an aneurism; all "compressible" tumours are not necessarily aneurismal. This very day, we have ourselves handled two abscesses of this character: one in the axilla, the other in the groin; both occurring in cachectic strumous subjects; one acute, the other chronic; both surrounded by considerable induration, within which the fluctuating tumour receded upon pressure, and seemed to disappear. One we opened without mishap ; the other is reserved for a future opportunity, in deference to the wishes of the patient.

A more negative position has been taken up, and it has been asked, " If it was an abscess, what became of the pus? why did not the matter show itself by gushing forth from the incision?" Courteous disputant! Did you ever open an abscess through some thickness of parts and find a difficulty in persuading your patient that the result within the recipient vessel was not " all blood, and no matter ?" Have not the common occurrences of surgical practice long since convinced you, that it requires no great amount of blood by commixture to disguise and conceal no inconsiderable quantity of purulent fluid? Can you not readily imagine how a few ounces of thin pus (it was a chronic abscess in a strumous child,) must have quickly disappeared in the whirling eddy of a pool by the side of the big carotid ? And are you not now satisfied that, supposing the case to have been as Mr. Liston states it was, it would have been indeed remarkable, and almost inexplicable, had a fluid presenting any of the distinctive characters of pus, followed the withdrawal of the bistoury ?

Shifting again, it is asked, " How could so much matter be suddenly thrown into the general circulation, without entailing the most disastrous consequences?" We reply, the pus-globules were few in number; the fluid was in its major part serous. And there is no proof of their *sudden* commixture with the general stream ; they may have been added so gradually (for the arterial opening was a small one,) as not necessarily to create aggravated constitutional irritation, or formation of purulent deposits within internal organs.

" Could the artery have been opened by the plunge of the bistoury ?" Certainly not. A mere glance at the relative position of the parts declares this idea extravagant, and the supposed fact impossible.

It is perhaps asked, why the artery was tied so near to the anonyma, at a point so palpably unfavorable to obliteration? There was plainly no choice; for the tumour had encroached so far on the lower part of the neck, as to render any more eligible site for deligation impracticable.

Do we wish for corroboration of our views in this case? we are not without supporters. M. Robert, a distinguished surgeon of France, in his treatise "Des Anévrysmes de la région sus-claviculaire," (Paris, 1841,) p. 81, observes " M. Liston, qui exerce avec éclat la chirurgie à Londres, a été accusé d'avoir ouvert un anévrisme de l'artère carotide qu'il aurait pris pour un abcès; mais les détails récents publiés par ce praticien établissent qu'il s'agissait bien dans ce cas, comme dans le précédent, d'un véritable abcès développé autour de l'artère carotide, dont les parois avaient été ramollies et érodées."

Some of the best surgical authorities of this country, whose opinions we have had opportunities of hearing, agree in the judgment of M. Robert. Among these we may name Sir Philip Crampton, as having no doubt whatever of the accuracy of Mr. Liston's view of the case. Sir Philip has given us the particulars of a very analogous case which occurred to himself many years ago. The cavity of a venereal bubo was similarly converted into a false aneurism, by communication with the ulcerated femoral artery. He had to tie the external iliac, and we are not sure but it was the first case in which that vessel had been deligated on that side of St. George's channel. The man was under treatment for syphilis in the Lock Hospital, and had an open bubo. Sir Philip was called to him in consequence of his having suddenly lost a large quantity of blood from the opening (which was small) in the abscess at the groin. The bleeding had ceased, but a large and obscurely pulsating tumour, double the size of the original abscess, had formed in its place. The external iliac, as already observed, was tied, with fair prospect of success; but secondary bleeding came on about the usual time, and had the usual termination—death.

We hold then, on these grounds, the conduct of Mr. Liston, in connexion with the case in question, to have been in no way reprehensible. On the contrary, the profession is much indebted to him for his candid narrative, and for the valuable instruction which it is made to bear. We see that even the most experienced, skilful, and dexterous surgeon may be deceived in diagnosis, and so be led into serious practical error. We are more than ever persuaded of the claim and necessity for all care and precaution in the examination of tumours, ere their character is definitely determined and their active treatment thereupon commenced: we are more than ever strengthened in the salutary dread of leaving an increasing abscess, whether chronic or acute, in continued juxtaposition with important vascular tissue: and we have been introduced to, and made acquainted with, a new form or variety of false aneurism, of the greatest pathological interest, and of the highest practical importance.

Other cases are quoted by Mr. Liston illustrative of how the aneurismal accession is made to the abscess, from the experience of Professor Syme, Dr. Craigie, Professor Fergusson, Sir James Macgregor, M. Breschet, Mr. Quain, &c. But these, and the object to which they tend, admitting of new dispute, we need not enter on their details. Suffice it to say, that they are all most interesting as well as apposite and conclusive.

The eminent author of this paper, we are well aware, requires no extrinsic aid to succour his professional fame and reputation. We have made the foregoing observations with no such object in view, but simply in justice to ourselves as impartial journalists, and in fulfilment of a solemn duty we owe our readers in all such matters, to "nothing extenuate, nor set down aught in malice."

---

## Figures and Tables

**Figure f1:**
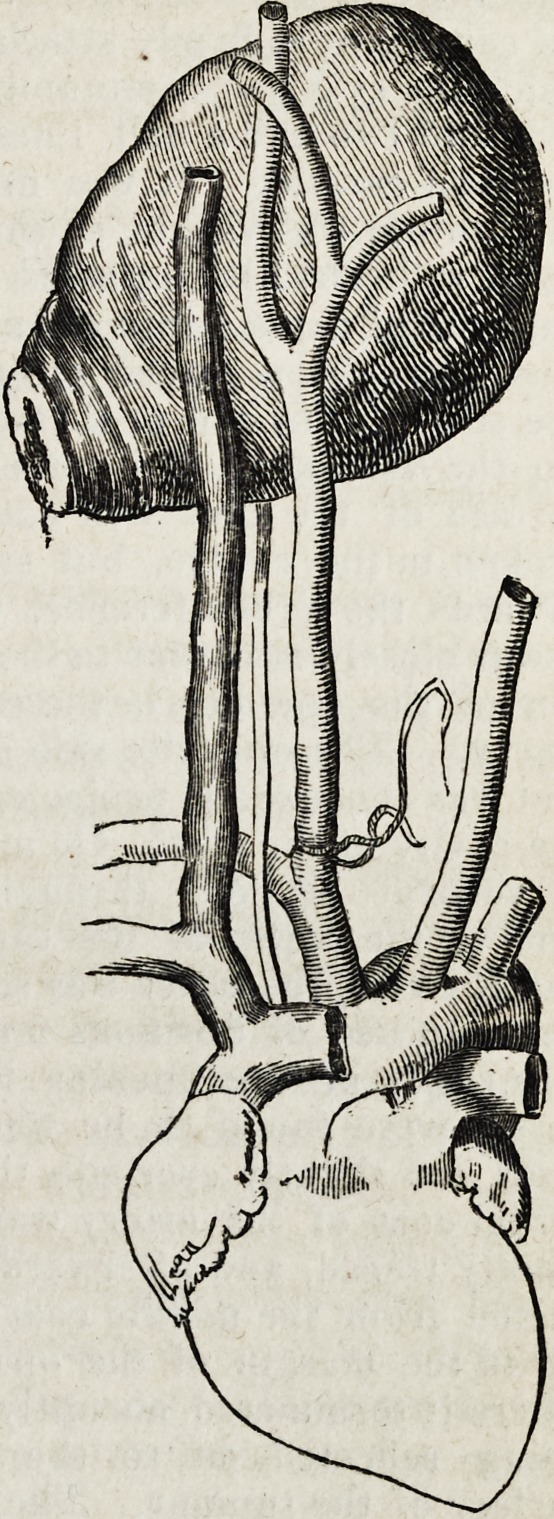


**Figure f2:**